# Experimental Analysis and Optimization Approach of Self-Clocked Rate Adaptation for Multimedia Congestion Control Algorithm in Emulated 5G Environment

**DOI:** 10.3390/s23229148

**Published:** 2023-11-13

**Authors:** Haider Dhia Zubaydi, Ahmed Samir Jagmagji, Sándor Molnár

**Affiliations:** 1 Department of Telecommunications and Media Informatics, Faculty of Electrical Engineering and Informatics, Budapest University of Technology and Economics, Műegyetem rkp. 3., H-1111 Budapest, Hungary; ahmedsa@tmit.bme.hu (A.S.J.); molnar@tmit.bme.hu (S.M.); 2 Department of Computer Engineering, College of Engineering, University of Mosul, Mosul 41002, Iraq

**Keywords:** congestion control, conversational video, experimental analysis, optimization, rate adaptation, self-clocked, WebRTC

## Abstract

The congestion problem has driven many researchers to address it, among other networking issues. In a packet-switched network, congestion is essential; it leads to a high response time to deliver packets due to heavy traffic, which eventually causes packet loss. Hence, congestion control mechanisms are utilized to prevent such cases. Several interesting algorithms are proposed to focus on this dilemma, such as the Self-Clocked Rate Adaptation for Multimedia (SCReAM) designed for interactive real-time video streaming applications. One of the main issues of SCReAM is the high design complexity due to the large size of its documentation and coding. Furthermore, there is a considerable number of parameters that can be adjusted to accomplish the desired performance. This study proposes a guided parameters’ tuning approach to assess and optimize the SCReAM algorithm in an emulated 5G environment through a detailed exploration of its parameters. The proposed approach consists of three phases, namely, the initialization phase, the standalone experimentation phase, and the hybrid experimentation phase. In the first phase, we illustrate the method of initializing and implementing the environment, followed by specifying the investigated parameters’ settings, testing, and validation. The second phase aims to investigate SCReAM parameters in isolation to identify the effect on the performance in relation to network queue delay, smoothed Round Trip Time (sRTT), and throughput. The final phase discusses the possibility of achieving the optimum performance by combining various sets to provide researchers with clear and explicit guidelines to establish an adequate SCReAM behavior for the desired application. To the best of our knowledge, this is the first study that proposes a preliminary and comprehensive analysis of the SCReAM algorithm. Based on the proposed approach, when L4S/ECN is disabled, we reduced the network queue delay by 63.36% and increased the network throughput by 48.6% as compared to the results generated by the original design. In L4S/ECN-enabled mode, the network queue delay is reduced by 16.17% while the network throughput increased by 93%.

## 1. Introduction

Because of the rate adaptation of Hypertext Transfer Protocol (HTTP) adaptive streaming, the quality of the video supplied to the end user will vary over time depending on the overall network environment. Video-related applications were responsible for over three-quarters of global mobile data transmission in 2021. With a similar video traffic growth, network operators must carefully and optimally utilize the existing video streaming resources and maintain an adequate Quality of Experience (QoE) for its users [1,2]. As the digital infrastructure grows with millions of users, networks suffer packet loss, radio frequency congestion, latency, and undesirable re-transmissions. Today, most real-time solutions demand high-speed data transfer, contributing to network saturation and unresponsiveness. Such demands lead to degradable data transmission and, eventually, network congestion. Several mechanisms must be considered to preserve efficient network performance and reduce congestion probability. Congestion control is indispensable in extending the network’s lifetime and improving its performance [3].

Congestion can be unpredictable, which leads to a loss of precious information in the network [4]. Therefore, congestion must be noticed to stop performance from degrading and the network from collapsing. In addition, congestion can be caused by suspicious events or undesirable actions on the network [5]. Even though the Internet Engineering Task Force (IETF) has made tremendous efforts to standardize WebRTC for real-time solutions, such as the Real-Time Transport Protocol (RTP), The industry demonstrated a hesitancy toward adopting standardized protocols, instead, opting for proprietary protocols and algorithms in its products. However, this approach has resulted in the limitation of interoperability among services [6].

Due to the massive rise in network traffic, network congestion is one of today’s most critical issues. Network congestion occurs whenever nodes and links are overloaded or if the transmitted bitrate is greater than the available bitrate over a dedicated transmission path. This leads to a situation that affects the performance of the network and the Quality of Service (QoS). Over time, transmission delay increases because of the subnets’ links overload, which causes a decrease in network performance [7]. Applications diffused over the network should apply congestion control to gain performance robustness and avoid congestion collapse. Nowadays, congestion control algorithms are a crucial component of any network structure to reduce any consequence on the users’ experience. One essential and efficient congestion control algorithm, Self-Clocked Rate Adaptation for Multimedia (SCReAM) [8], was presented in 2014 for the video conversation of LTE networks. Then, it was standardized in 2017 [9].

In our previous research, we explored the possibility of implementing SCReAM in an emulated 5G environment [10]. Then, we proposed an approach to achieve the best possible streaming in an emulated 5G environment based on a congestion window [11]. In this research, we propose a guided parameters’ tuning approach to achieve the optimum performance of the SCReAM algorithm through an optimal parameter set in an emulated 5G environment in terms of network queue delay, sRTT, and network throughput. This research employs our previous works and offers an extended and comprehensive approach to address the specified metrics. Extensive experiments are performed to identify the effect of each parameter in isolation. Later, in the hybrid experimentation phase, various sets of parameters are incorporated to achieve the highest possible performance. It is important to mention that the main goal of this paper is to focus on QoS metrics; QoE evaluation is outside the scope of this study. Moreover, we are not aiming to provide a global general performance analysis; our results and conclusions are based on our implemented scenarios.

This paper is structured as follows: Section 2 reviews the related work that introduced congestion control algorithms and highlights the approach and the design of the SCReAM congestion control algorithm. Section 3 presents the methodology used in our experiments. Section 4 discusses the results obtained, followed by a detailed discussion. Finally, Section 5 discusses the conclusion and future works.

## 2. Overview

### 2.1. Related Work

One of the most essential modified versions of TCP’s congestion control mechanism for TCP connections with wide congestion windows is HighSpeed TCP (HSTCP) [12]. HSTCP alters the TCP response function on high-capacity links to achieve better performance. It relies on the Additive Increase Multiplicative Decrease (AIMD) algorithm for increasing and decreasing the functions of the congestion window’s current value, resulting in an adaptive and more or less scalable technique.

A good example of implementing the Multiplicative Increase Multiplicative Decrease (MIMD) mechanisms for TCP is Scalable TCP (STCP) [13]. STCP is a proposed and efficient transport protocol for high-speed networks where the Multiplicative Increase and Multiplicative Decrease techniques ensure the protocol’s scalability. When an acknowledgment is received, a constant parameter is used to expand the congestion window. However, the congestion window is decreased in a multiplicative way when packet losses occur.

Further research into delay-based ideas has led to FAST TCP [14], which depends on the TCP Vegas approach [15] to handling congestion. Congestion handling is based on the delay signal, which aims to achieve high bandwidth and low queuing delay utilization, leading to great Bandwidth-Delay product (BDP) paths [16]. FAST TCP attempts to hold a limited number of packets in the queue of a bottleneck by utilizing the base Round Trip Time (RTT) value extracted from the observed minimum RTT and the current average RTT. It also modifies the CWND every fixed period instead of updating it to one Maximum Segment Size (MSS) in each RTT interval.

The combination of the loss-based and delay-based approaches appears in TCP-Illinois [17], which utilizes loss as the initial congestion signal and delay as the second. The protocol implements an AIMD technique by adjusting the increase and decrease parameters according to the observed queuing delay. Furthermore, several congestion control protocols depend on Explicit Congestion Notification (ECN) rather than implicit congestion signals. Some of the signals used within the implicit congestion signals are packet loss or delay. These techniques rely on network routers for congestion control, which also requires the routers to be modified [18]. One of the most notable protocols, which extends the ECN [19], is the eXplicit Control Protocol (XCP) [20]. Routers supporting the XCP technique report the bottleneck congestion’s degree to the sender instead of using an ECN one-bit congestion signal. Additionally, XCP separates fairness control and load control.

The Signaling Transport Control Protocol (SCTP) [21] is a standardized protocol for signaling that was established by the IETF and has expanded into a general-purpose protocol. Similar to TCP, this protocol is implemented within the kernel of the operating system. It provides a dependable, point-to-point, and connection-oriented means of transporting data over IP networks. SCTP was deliberately developed to be compatible with TCP, incorporating similar retransmission techniques, congestion control (based on window size), and error detection. However, SCTP also has other unique features, including multistreaming and multihoming. The process of establishing and terminating a connection varies slightly between TCP in order to enhance operational efficiency and security. The utilization of a cookie mechanism in the initial four-way handshake serves as a preventive measure against masquerade and SYN flooding attacks. Additionally, it prevents the occurrence of half-closed connections, where one endpoint retains the ability to transmit data.

Low Extra Delay Background Transport (LEDBAT) [22] is a substantial Low-Priority Congestion Control (LPCC) algorithm that is implemented in different transfer applications such as software updates and peer-to-peer file transfer [23]. LEDBAT aims to maintain the forward queuing delay as low as possible to reduce interference with other flows utilized by latency-sensitive applications such as video games, media players, and Voice over IP (VoIP). LEDBAT observes queuing delay; any increase beyond a threshold indicates network congestion. It utilizes One-Way Delay (OWD) calculations instead of RTT to bypass any fluctuations in the delay in the inverse path.

LEDBAT uses a threshold (default 100ms) for the predefined target to set the queuing delay. It increases or decreases the CWND in accordance with the differences between the estimated and target queue delay in case of no packet loss. At the same time, it acts as the standard TCP and halves the CWND if the loss is discovered. To ensure that LEDBAT is less aggressive than standard TCP, a scale referred to as gain (G) specifies the CWND growth/drop rate and must not be greater than one. OWD is measured for every transmitted packet to respond as quickly and accurately as possible to any variations in the delay. LEDBAT keeps a record (ten entries as a default) of the base OWD and every element describing the minimum OWD (OWDmin) measured for a single minute. The base OWD represents the least value of the ten entries list. The mentioned record is deployed later to decrease the sudden change effect in base OWD assessment due to the delay in the acknowledgment ACK and re-routing problems.

Several studies assessed the LEDBAT performance and discovered that it shows a delay increase and experiences some issues with improper propagation delay estimation [24,25,26]. Additionally, a study showed that the LEDBAT flows act too aggressively if their parameters are chosen randomly in AQM environments. This behavior is due to the loss signal controlling the CWND backs-off because the delay will never reach the LEDBAT target delay [27].

LEDBAT++ [28] is an improved algorithm introduced to solve some of the weaknesses in the LEDBAT congestion control algorithm. LEDBAT++ expands the LEDBAT functionalities by adding some modifications, such as reduced congestion window gain, modified slow-start, multiplicative decrease, and periodic slowdowns. By adding these enhancements, LEDBAT++ will address the known issues with the LEDBAT algorithm, including latency drift, latecomer advantage, and inter-LEDBAT fairness.

rLEDBAT [29], a collection of techniques that allow the implementation of a less-than-best-effort (LBE) congestion control algorithm for TCP at the recipient side. rLEDBAT basically consists of three different mechanisms, including a mechanism that determines the delay of the packet; it is responsible for packet loss detection, and a mechanism that handles the receive window to estimate the recipient’s rate. The sender’s rate is controlled by rLEDBAT recipient through the TCP header’s announced Recipient Window. rLEDBAT assumes that the transmitter is a standard TCP transmitter, so it does not need any further changes in the TCP sender. By optimizing the overall resource allocation, the enhancement of LBE traffic utilization and the advantages of using the global Internet can be facilitated through the novel use cases and deployment models using rLEDBAT.

The Prague [30] Congestion Control Scheme is an algorithm inherited from the Data Center Transmission Control Protocol (DCTCP) and modified to be utilized for Internet traffic by applying the Prague L4S constraints. It is designed to achieve uniformly low queuing delays and full throughput over network links, providing Low-Latency Low-Loss Scalable Throughput (L4S) support at the bottleneck. AIMD is used by the Prague Congestion Control Scheme, where it expands its window until it reaches a value that can be detected by an ECN mark (or loss) and then proceeds in a sequential notched form.

In [31], the authors explained the interoperability issues between LEDBAT++ and the Bottleneck Bandwidth and Round trip propagation time (BBR) congestion control algorithms that lead to unexpected behavior. Under certain conditions, LEDBAT++ does not proceed to BBRv1 and BBRv2. They performed several experiments to figure out the LEDBAT++ BBR relations. They found that when two TCP connections (BBRv1 and LEDBAT++) with the same RTT compete for the capacity of the bottleneck link, using a buffer large enough to generate a queuing delay at least equal to LEDBAT++’s target, the following observations are assembled: When the base RTTs are more significant than the target queue delay (T), LEDBAT++ acts as a scavenger transport and gives up before BBRv1. When the base RTTs are smaller than T, LEDBAT++ grabs a significant amount of capacity. The other experiment used BBRv2 instead of BBRv1, and the difference was that the LEDBAT++ conducts similar to a scavenger transport and gives up before BBRv2 for the first experiment conditions. When the base RTTs are smaller than T, BBRv2 gives up before LEDBAT++, conducting just opposite to expectations.

QUIC [32] is a transport layer protocol that offers improved congestion control by adapting the TCP-Friendly Rate Control (TFRC) algorithm. Additionally, loss-based mechanisms are employed to identify any loss caused by the congestion. Furthermore, QUIC includes the following:

- A congestion window similar to the method used in TCP.
- A pacing mechanism to control the inter-packet arrival time and sending rate.
- ACK frequency by implementing Selective Acknowledgement (SACK) to provide feedback on the received packets.
- Congestion feedback through the utilization of ECN techniques.
- Congestion avoidance, such as AIMD.

### 2.2. SCReAM Algorithm

An efficient congestion control algorithm called SCReAM was introduced in 2014 [8] and standardized in 2017 RFC8298 [9]. RFC8298 “Self-Clocked Rate Adaptation for Multimedia” presents an outline of the rate adaptation algorithm explicitly designed for conversational media services. SCReAM is a rate adaptation congestion control algorithm that integrates loss-based and delay-based algorithms to construct a hybrid model for LTE networks. It uses the idea of packet conservation to control network congestion and adjusts the network changes to evaluate variables such as rate and queuing delay [33]. Depending on the evaluations, SCReAM acclimates to the changes by altering the network parameters to gain optimum performance. Furthermore, it decreases the deviations of short-term delay through an efficient congestion window computation mechanism. SCReAM is the basis for the rate adaptation concept and other techniques derived from the TCP-friendly window-based [34] and LEDBAT. It also benefits from the self-clocking feature by obtaining a shorter timescale operation. Although SCReAM was initially designed for WebRTC, it can be applied in various applications where RTP streams are essential. Due to several advantages, including its better performance over the other delay-based mentioned algorithms, we decided to choose SCReAM as the base for our experiments, evaluations, and analyses [10].

**Figure 1:**
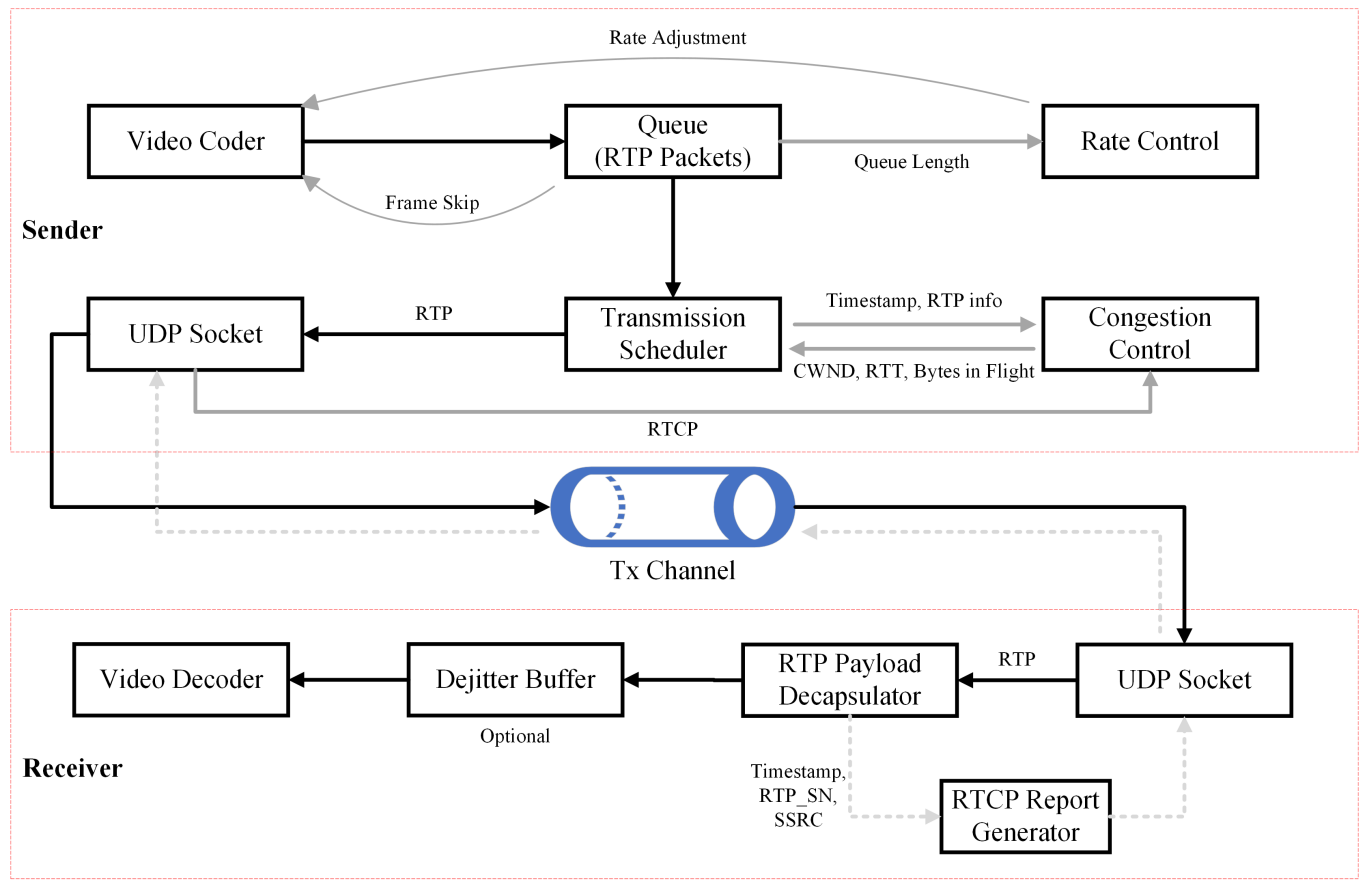
SCReAM architecture (a single media source design) [35].

A full description of SCReAM’s components and the process of generating the frames, encoding in the sender, and decoding in the receiver are shown in Figure 1. The main components of SCReAM are network congestion control, media rate control, and sender transmission control. Other secondary components of the sender are the UDP socket and RTP packets queue, while the receiver contains an RTP payload decapsulator, de-jitter buffer (optional), and video decoder. Since the crucial functions of the SCReAM congestion control algorithm are implemented on the sender side, we will focus on describing its components.

The rate control unit adjusts the media bitrate (referred to as target bitrate excluding RTP and Forward Error Correction (FEC) overhead bitrates) by comparing it with a pre-determined threshold based on RTP queue size. The amount of transmitted data is determined in the congestion window, which is related to the bytes in flight described as the possible size of the data being handled inside the network at a specific time interval (specified based on the network congestion control unit). The process of determining the amount of transmitted data is handled by the sender transmission control unit (transmission scheduler). Estimated link throughput and packet size are the main factors affecting the transmitted data size. The receiver specifies the congestion window size by generating feedback to the transmitter through the RTCP report generator unit.

Each component in SCReAM’s design is responsible for a specific task starting from the initial frames until feedback is received. In Figure 1, the media frames undergo the encoding process and are subsequently delivered to the RTP queue. The media rate adaptation mechanism adjusts its behavior based on the size of the RTP queue and determines a desired rate for the media encoder. For multiple flows originating from each RTP queue, the sender transmission controller selects RTP packets from the RTP queue in accordance with a predetermined priority order or a round-robin manner. It is also responsible for sending the RTP packets to the UDP socket during multiple flows. In the typical scenario, the media must undergo transmission control to ensure that the amount of data sent at any given moment, known as the bytes in flight, remains below the congestion window. Later, the packets are forwarded to the receiver. Finally, the sender transmission control and network congestion control units exchange network information related to the CWND and bytes in flight.

SCReAM is more suitable than rate-based algorithms since it is designed with the self-clocking principle, which allows the algorithm to run over shorter periods (1 RTT). Additionally, it evolves computation of the CWND over a longer timescale. However, SCReAM’s design is complex because of the complicated documentation and coding. Furthermore, several parameters are included in the algorithm, assigned with specific values in the initial examination. Thus, the most crucial parameters are selected and investigated in this study, as explained in the following section.

SCReAM can be operated in two working modes: Low-Latency Low-Loss Scalable Throughput/Explicit Congestion Notification (L4S/ECN)-enabled and -disabled. L4S is proposed to work with multimedia traffic applications, which require a low delay [36]. SCReAM can take advantage of L4S, which aims to reduce the transmitting rate proportionally to the ECN-marked RTP packets fraction, to help minimize the network queue delay. SCReAM also supports the classic ECN that aims to reduce the end-to-end delay when one or more ECN-marked RTP packet is in one RTT [37]. Note that ECN has to be supported by the network and the hosts to be successfully implemented by SCReAM or other congestion control algorithms. However, it is not required that we apply the L4S/ECN techniques for the basic congestion control functionality in SCReAM, as we previously showed in [10].

L4S is a technology developed to decrease queue delay issues, providing low latency to Internet Protocol (IP) streams with a high throughput performance. L4S depends on ECN to achieve this purpose. ECN is a technique that labels packets to indicate network congestion and prevent packets from being dropped. The congestion signals are managed at both the sender and receiver with the help of scalable congestion control algorithms [36]. ECN helps to minimize end-to-end latency. Another term used with the ECN is ECN Capable Transport (ECT), which is a codepoint to notify the network nodes that the packet is enabled to support ECN marking. The most important benefits of applying the ECN marking are improving the throughput, reducing head-of-line blocking, and minimizing the probability of Re-transmission Timeout (RTO) expiry [38].

L4S is activated and alters the IP header’s ECN bits whenever the network nodes’ queues grow above a specific threshold. The changing of the ECN bits to notify congestion is named marking. L4S is responsible for activating the AQM to let the network nodes control the packet congestion notification experience transmitted from a sender to a receiver using ECN bits in the IP header. There are some steps for implementing communication with the support of L4S. First, the transmitter marks L4S support by using specific ECN codepoints. Then, the network nodes identify the L4S packet. The L4S packet is marked if congestion occurs by altering the ECN bits to distinguish CE. Whenever the packet arrives at the recipient with the ECN bits marked as Congestion Experience (CE), the recipient informs the transmitter about the congestion. Finally, the transmitter informs about the congestion and starts to reduce the transmitting rate [36]. There are specific states needed to perform the L4S support, such as:

1. The receiver should be able to detect the CE codepoint and inform the transmitter of the congestion.
2. The transmitter adjusts the sending rate to stop the network from congestion. Each node must recognize an L4S packet from a not-L4S packet for successful congestion management.
3. The not-L4S packet (classic packet) should be processed as normal, while L4S packets must have a dedicated queue to mark the CE packets whenever the queue begins to grow.
4. The network nodes should have two queues (for the L4S traffic and the not-L4S traffic). The codepoint name and meaning of L4S/ECN bits are demonstrated in Table 1.

When L4S/ECN mode is enabled, the SCReAM algorithm activates CE by triggering the ECN-CE marking event. Note that CE events on the sending side are checked at least every 30 ms. The algorithm then takes the current timestamp and sets its value as a timer for the last loss and the last CE events. The loss event is specified when one or more RTP packets are reported as lost in one RTT, and, in that case, lost RTP packets must be neglected for a full sRTT. SCReAM detects the presence of an ECN event when it detects an increase in the n_ECN counter, the cumulative number of ECN-CE-marked packets in the last receiver’s feedback report.

Once an ECN event is detected, the n_ECN counters are ignored to maintain sRTT and help limit CWND reductions to at most one per Round Trip Time. Afterward, SCReAM checks for the L4S mode; if enabled, it calculates a congestion scaling factor that depends on the proportion of ECN-marked packets. Hence, SCReAM quickly reduces network throughput, which reduces the CWND sufficiently. Next, the sender checks whether the event is a loss or ECN-CE and activates the loss event flag or the ECN-CE event flag. Finally, the target bitrate is updated according to a specific ratio, and the transmission rate is reduced as described in [9,37].

It is essential to highlight critical aspects such as generalization and transferability. Regarding the first term, SCReAM’s adaptation to suit various use cases can be easily made due to the underlying mechanisms and principles. For example, a self-clocking principle implemented at the sender, a congestion control algorithm that calculates the packet loss rate and RTT, a rate adaptation technique to adjust the sending rate based on congestion level and network capacity, and a feedback mechanism that relies on the receiver that indicates any congestion event. Such advantages entitle SCReAM to be viewed as a versatile algorithm for optimized WebRTC communications.

Regarding transferability, the original design of SCReAM is intended to be built in diverse real-time multimedia systems, applications, and environments. The utilization of SCReAM takes place in interactive multimedia services, online gaming, video streaming, and video conferencing. It can be implemented in wired or wireless networks and various technologies beyond Wi-Fi, LTE (original design), and 5G. Such flexible design enables seamless integration in different communication systems and utilization in distinct scenarios. Furthermore, the integration can be efficiently executed in current networking infrastructures because SCReAM relies on standard IP mechanisms while adhering to and leveraging the widely supported protocols and mechanisms. Widespread adoption is facilitated due to SCReAM’s compatibility and adaptability that allows and ensures smoother user experience, optimized video quality, and improved congestion control performance.

## 3. Methodology

**Figure 2:**
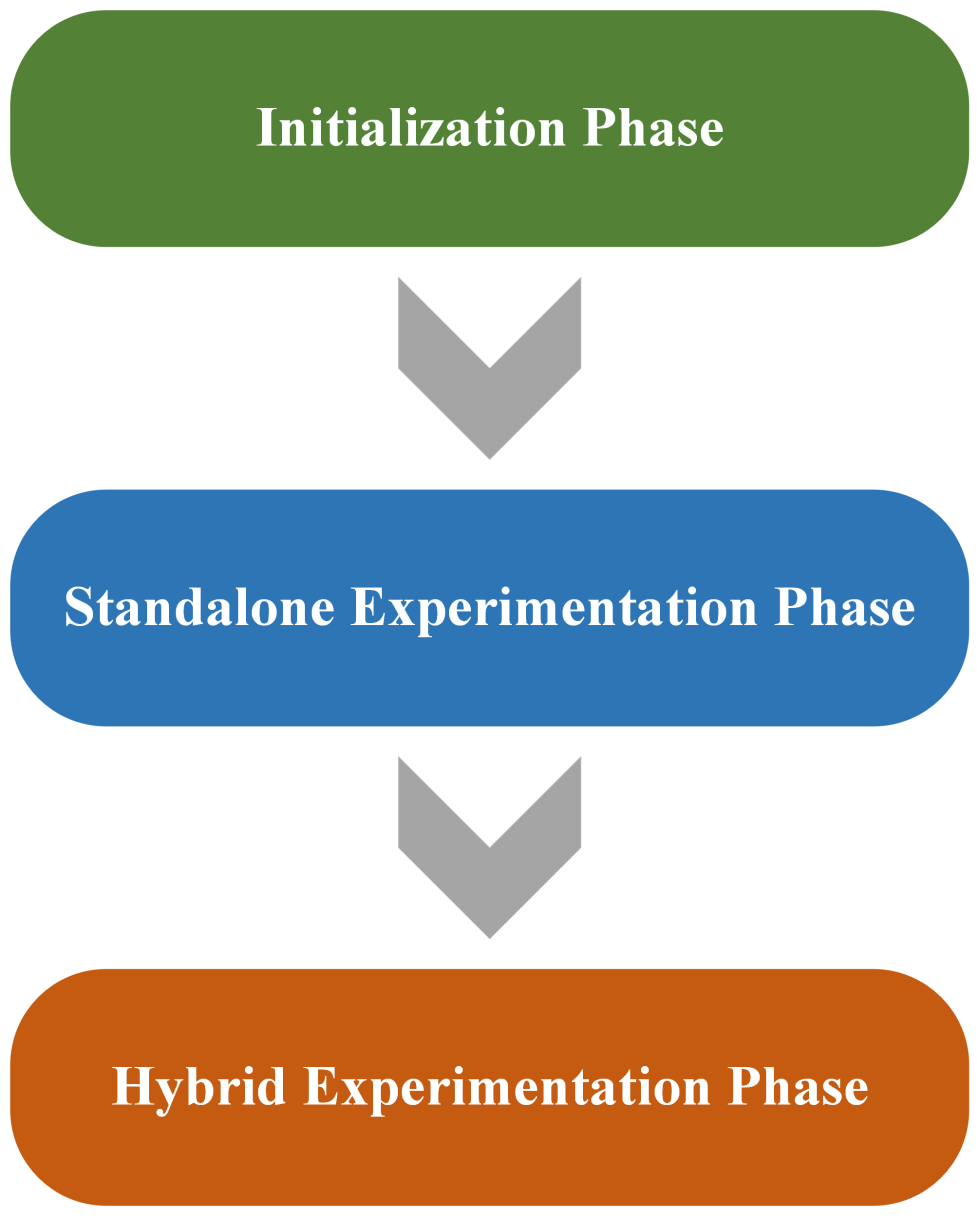
Design phases.

As illustrated in Figure 2, our methodology consists of three main phases: the initialization phase, the standalone experimentation phase, and the hybrid experimentation phase. Each phase is discussed in detail in the following subsections:

### 3.1. Initialization Phase

**Figure 3:**
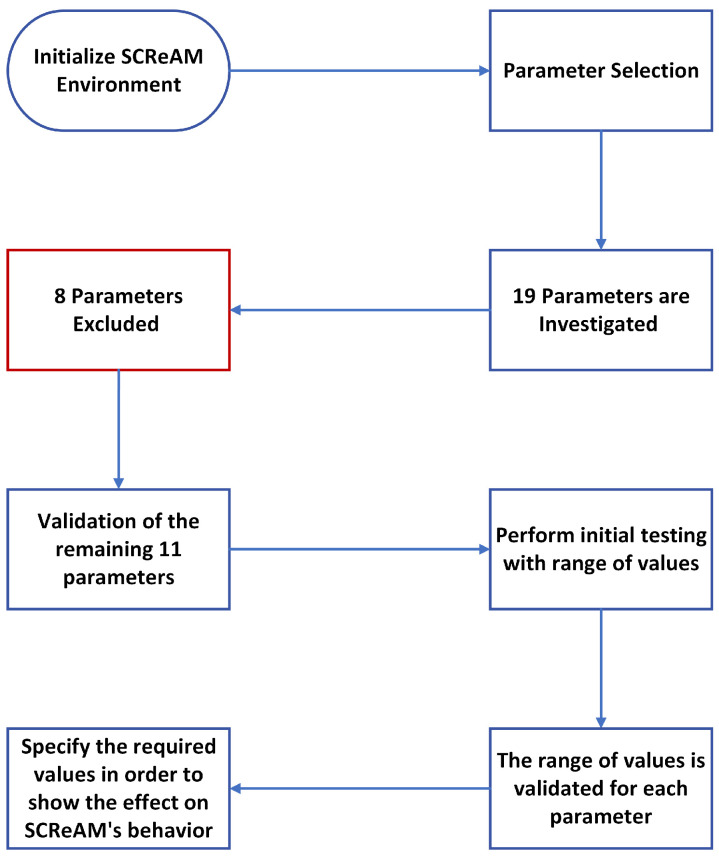
Initialization phase stages.

This phase has two parts; the first part presents how the SCReAM algorithm environment is initialized and implemented. The second part concerns the investigated parameters’ setting, testing, and validation. Figure 3 illustrates the detailed stages performed in this phase. We can run SCReAM in two methods [10]. The first method uses the Windows-based test application using Visual Studio software (v17.7.4), which we used in our experiments. Using this setup, SCReAM implements a single sender and receiver where the functions described in Figure 1 are performed. The other method depends on the Linux-based BW test application [37], which we implemented when we tested the SCReAM for the first time. The method we used in our experiments includes several C++ codes comprising sender, receiver, and multiple supporting classes (NetQueue, Video Encoder, and RTPQueue) and the coordinator code (scream_v_a), which is responsible for controlling and linking all of the codes together.

**Figure 4:**
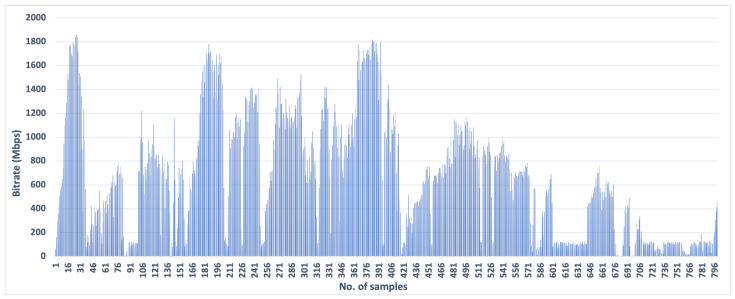
Emulated 5G dataset traces used for the network shaping [10,11].

We tested the performance of SCReAM in the emulated 5G environment using the dataset traces obtained from [39,40], shown in Figure 4. The dataset used in our experiments is extracted from a measurement study of commercial mmWave 5G services in a U.S. city (Minneapolis). It concentrates on the throughput perceived by applications operating on User Equipment (UE). The data of this dataset is extracted from real-time 5G measurements. The dataset size is 38,632 GBs downloaded over a 5G network captured by walking over 331 km and driving over 132 km in 6 months. We tested several traces to ensure that SCReAM functions correctly because the bandwidth contains rapid changes over small intervals. The bitrate of the 5G traces is in the range from 0 to 1860 Mbps. We utilized the 5G dataset in SCReAM’s code to control the transmitted data (available bandwidth) emulating the 5G environment. The 5G trace file has 800 samples, but we set the algorithm to reuse the trace file more than once to cover the whole simulation time (100 s).

The second part of this phase includes selecting the possible parameters to analyze and optimize the performance of SCReAM. Many parameters were investigated based on RFC8298 [9] and the coding. Eventually, a total of 22 parameters were initially investigated. Later, each parameter is studied in detail to identify its possible effect. Furthermore, three parameters were excluded due to their insignificant roles in our environment.

A total of 1536 experiments are performed with the remaining 19 parameters to ensure that the included parameters are appropriate to our goals. Thus, each parameter must be selected precisely. After experimenting with the remaining parameters, we realized that 11 parameters fit our requirements (based on our scenarios). The reason behind our selections is that we aim to address specific parameters that considerably affect network latency and throughput; these metrics are considered the most critical when evaluating any networking architecture. The original design of SCReAM includes many output metrics, such as packet loss, congestion window, bytes in flight, etc., which are irrelevant to this research. The excluded 8 parameters did not show any impact on network latency and throughput. They are designed to affect one or more metrics out of this study’s scope. Table 2 depicts the details of the selected parameters. The validation process is accomplished by estimating the algorithm’s output based on the mathematical model used in SCReAM. The selected parameters were validated by comparing the estimated and the actual output.

In order to conduct our experiments properly, a range of values must be specified for each parameter to illustrate each parameter’s effect on overall performance clearly. We started our experiments with a wide range of values for each parameter. After a certain number of experiments, the range narrowed down. Narrowing the range is triggered only when there is an unnoticeable change in the performance, thus eliminating the margin values that produce the same performance metrics’ values. We aimed to keep the default value of each parameter as the middle value in the range to provide a clear understanding of performance changes before and after it. The default and range of values for each parameter are shown in Table 2. It is essential to mention that for $TB_{max}$, the default value for the maximum target bitrate is 20 Mbps, which was set initially in the algorithm; however, since we are implementing the algorithm to run at high speeds in our experiments, we eventually set the default value to 100 Mbps.

### 3.2. Standalone Experimentation Phase

In the second phase, we aim to provide the algorithm for exploring each parameter’s effect. As shown in Algorithm 1, multiple variables are defined and used to construct the algorithm as shown below:

- *i*: parameter number.
- $i_{Max}$: the maximum number of parameters for the experiments.
- *A*: a position in array of parameter *i*.
- P(A): an array containing the set of values for parameter *i*.
- $V_{i}$: a value of the specified parameter.
- NoS: (number of samples): used as a counter that represents the number of samples to calculate the average value of the output metrics.
- NetQD, sRTT, BW: network queue delay, sRTT, and network throughput, respectively.
- $NetQD_{Avg}$, $sRTT_{Avg}$, $BW_{Avg}$: the average value of network queue delay, sRTT, and network throughput, respectively.
- Variables with ${}^{\prime }$ sign represent the newly generated value of that variable.
- time: emulation of real-time clock calculated within the algorithm.

**Algorithm 1** Generating Separate Sets of Values for Each Parameter**Input:** *i*, $i_{Max}$, *A*, P(A), $V_{i}$, NoS, time, NetQD, sRTT, BW**Output:** $NetQD_{Avg}$, $sRTT_{Avg}$, $BW_{Avg}$1:**if** (isL4S=True) **then**2:   $i_{Max}\leftarrow 11$;3:**else**4:   $i_{Max}\leftarrow 9$;5:**end if**6:**while** $i\leq i_{Max}$ **do**7:   A←0;8:   **if** $V_{i}\leq MAX\left(P\left(A\right)\right)$ **then**9:     **if** NoS<2000andtime≤100 **then**10:        $NetQD\leftarrow NetQD+NetQD^{\prime }$;11:        $sRTT\leftarrow sRTT+sRTT^{\prime }$;12:        $BW\leftarrow BW+BW^{\prime }$;13:        Increment NoS;14:        Increment time;15:     **else**16:        $NetQD_{Avg}\leftarrow NetQD/NoS$;17:        $sRTT_{Avg}\leftarrow sRTT/NoS$;18:        $BW_{Avg}\leftarrow BW/NoS$;19:        NoS←0;20:        Increment *A*;21:     **end if**22:   **else**23:     Increment i;24:   **end if**25:**end while**

Since our experiments are performed in two L4S modes, the first step of the algorithm is to check the status of L4S. When L4S is enabled, two parameters do not affect the algorithm’s performance; thus, these parameters are excluded. Our algorithm checks whether *i* is larger than $i_{Max}$ to ensure we have covered all parameters. Our algorithm applies the possible values within the selected range for a specific parameter, and each value is experimented with separately. For example, since ten values are selected for each parameter, when $i_{Max}=11$ (L4S disabled), the number of experiments is 110. When $i_{Max}=9$ (L4S enabled), the number of experiments is 90; therefore, the total number of experiments performed in this phase is 200. In each experiment, the number of calculated samples for each metric is 2000 because we previously set the frame rate to be 50 frames per second (fps), and the simulation time is 100 s. When 2000 samples are collected or the simulation time reaches 100 s, the average network queue delay, sRTT, and network throughput are calculated by dividing NetQD, sRTT, and BW by NoS.

### 3.3. Hybrid Experimentation Phase

The final phase is called hybrid since the parameters are mixed. The issue we encountered relies on the total number of experiments because applying all possible values within the selected range results in a vast number of experiments, approximately $11^{10}$. Thus, to optimize SCReAM, only one pair of values (two iterations) for each parameter is selected. However, two parameters (i.e., minimum and maximum bitrates) still conflict with the previous statement since modifying their values in this phase does not impose reasonable results. For example, increasing the minimum bitrate from 2 to 10 Mbps is possible, which results in better performance; however, it does not reflect an actual real-time environment performance. Hence, the minimum bitrate will be fixed at 2 Mbps while the maximum bitrate is 100 Mbps, allowing an effective algorithm’s performance when it operates at low and high loads.

The pair values are selected based on the overall performance of SCReAM for all metrics. For example, even when other pair values can further reduce the network delay, throughput is affected negatively. Eventually, the hybrid experimentation included nine parameters, resulting in 1024 experiments divided into 512 + 512 when L4S is disabled and enabled. Accordingly, the pair values selected for this phase are shown in Table 3.

We modeled an algorithm to generate the mixed sets based on the previously mentioned details. As depicted in Algorithm 2, 512 unique sets are generated for each experiment. Multiple counters ($C_{i}$) are used to ensure that only two values are included for each parameter. $P_{i}\left(C_{i}\right)$ represents the array that contains the pair values for parameter *i*. $V_{i}\left(C_{i}\right)$ is related to a specific value inside the array. Each while in the algorithm represents two iterations starting from $C_{i}$ to $C_{i+j}$ where *j* represents the last parameter. Algorithm 2 reflects the main concept used to generate the sets; however, the actual implementation contains more iterations to reach a total of 512 unique sets, which is why $C_{i+j}$ is used. Finally, as previously performed, the average network queue delay, sRTT, and network throughput are calculated for each set.
**Algorithm 2** Generation of Mixed Parameters’ Sets**Input:** *i*, $i_{Max}$, $C_{i}$, $P_{i}\left(C_{i}\right)$, $V_{i}\left(C_{i}\right)$, NoS, time, NetQD, sRTT, BW**Output:** $NetQD_{Avg}$, $sRTT_{Avg}$, $BW_{Avg}$1:**if** (isL4S=True) **then**2:   $i_{Max}\leftarrow 9$;3:**else**4:   $i_{Max}\leftarrow 7$;5:**end if**6:**while** $C_{i}<2$ **do**7:   $P_{i}\left(C_{i}\right)\leftarrow V_{i}\left(C_{i}\right)$;8:   Increment $C_{i}$;9:   **while** $C_{i+1}<2$ **do**10:     $P_{i+1}\left(C_{i+1}\right)\leftarrow V_{i+1}\left(C_{i+1}\right)$;11:     Increment $C_{i+1}$;12:     **while** $C_{i+2}<2$ **do**13:        $P_{i+2}\left(C_{i+2}\right)\leftarrow V_{i+2}\left(C_{i+2}\right)$;14:        Increment $C_{i+2}$;15:        **while** $C_{i+3}<2$ **do**16:          $P_{i+3}\left(C_{i+3}\right)\leftarrow V_{i+3}\left(C_{i+3}\right)$;17:          Increment $C_{i+3}$;18:          **while** $C_{i+4}<2$ **do**19:             $P_{i+4}\left(C_{i+4}\right)\leftarrow V_{i+4}\left(C_{i+4}\right)$;20:             Increment $C_{i+4}$;21:             **while** $C_{i+j}<2$ **do**22:               $P_{i+j}\left(C_{i+j}\right)\leftarrow V_{i+j}\left(C_{i+j}\right)$;23:               Increment $C_{i+j}$;24:               **if** NoS<2000andtime≤100 **then**25:                  $NetQD\leftarrow NetQD+NetQD^{\prime }$;26:                  $sRTT\leftarrow sRTT+sRTT^{\prime }$;27:                  $BW\leftarrow BW+BW^{\prime }$;28:                  increment NoS;29:                  increment time;30:               **else**31:                  $NetQD_{Avg}\leftarrow NetQD/NoS$;32:                  $sRTT_{Avg}\leftarrow sRTT/NoS$;33:                  $BW_{Avg}\leftarrow BW/NoS$;34:                  NoS←0;35:                  time←0;36:               **end if**37:             **end while**38:             $C_{i+j}\leftarrow 0$;39:          **end while**40:          $C_{i+4}\leftarrow 0$;41:        **end while**42:        $C_{i+3}\leftarrow 0$;43:     **end while**44:     $C_{i+2}\leftarrow 0$;45:   **end while**46:   $C_{i+1}\leftarrow 0$;47:**end while**

The iterations of each parameter are presented in Table 4. The value of each parameter is specified based on a specified manner in every experiment. For example, parameter $P_{i}\left(C_{i}\right)$ changes once after 265 experiments while parameter $P_{i+1}\left(C_{i+1}\right)$ is modified every 128 experiments, and $P_{i+3}\left(C_{i+3}\right)$ changes every 64 experiments. Parameters 5 and 6 (minimum and maximum bitrates) are represented by a constant value line, as discussed previously, due to fixing their values. The last parameter, represented by $P_{i+j}\left(C_{i+j}\right)$, changes with each experiment.

## 4. Results

In this section, we present various experiments with newly investigated parameters, mentioned in RFC8298 [9], that aim to realize how each parameter affects the performance of SCReAM. The output of each experiment includes seven metrics: network queue delay, sRTT, CWND, bytes in flight, throughput, the scale of frames’ sizes, and total frames sent. These experiments are part of our work to create a reliable dataset for our future goals. We will focus on the essential network metrics, such as network queue delay, sRTT, and throughput. We measure the average value of the total simulation time (100 s) for each of the three metrics.

The network queue delay is the assessed queue delay of the entire network, which is the summation of the queue delay in each node in the network, computed by SCReAM. The network queue delay is measured at the Packet Data Convergence Protocol (PDCP) layer [36]. On the other hand, TCP implementations attempt to predict future Round Trip Times by sampling the behavior of packets sent over a connection and averaging those samples into a ‘‘smoothed’’ Round Trip Time estimate, which is referred to as the sRTT [41]. In SCReAM, the sRTT is calculated as follows:

(1) $$sRTT_{i}+1=\left(\alpha *sRTT_{i}\right)+\left(1-\alpha \right)*S_{i}$$

where $sRTT_{i}$ is the current estimate of the Round Trip Time, $sRTT_{i}+1$ is the newly computed value, α is a constant between 0 and 1 that controls how rapidly the SRTT adapts to change, and S is a sequence of round trip samples.

Section 4.1 refers to the results of each parameter in isolation, and Section 4.2 discusses the hybrid phase results:

### 4.1. Part 1

As discussed in the previous section, each parameter is defined within a specific margin based on our experiments to identify its effect on SCReAM’s performance. Due to the results’ size, combining the impact of all parameters in each metric is most appropriate to compare their actual values in each state. However, due to the difference between the parameters’ values, it is crucial to construct ten transition values for each parameter within the specified margins to visualize them properly. A transition value refers to a primary value representing several other close values (higher and lower). For example, the fifth transition value of RUS equals 10 Mbps, and we noticed that the results obtained from this value are close to the values in the range of 9–11 Mbps, which is the reason that the previous and next values are 8 Mbps and 12 Mbps, respectively. Thus, it can be realized that in the following figures, the *x*-axis contains ten values (1–10), where each value represents a transition for the investigated parameters.

The transition values are described in Table 5; bold values represent the default values. In each transition value, a distinct value is assigned for each parameter. The transition values are uniformly distributed (mostly) around the default value and in the range shown in Table 2. The default values of $TB_{min}$ and $TB_{max}$ are the minimum and maximum, respectively. Thus, values lower than 1 Mbps for $TB_{min}$ and higher than 100 Mbps for $TB_{max}$ do not indicate any changes in SCReAM’s performance. In addition, it is essential to mention that the last transition value of TRS (1.0) indicates that, in certain events (where the algorithm is required to reduce the target rate by 5%), the algorithm neglects the reduction.

#### 4.1.1. Network Queue Delay

**Figure 5:**
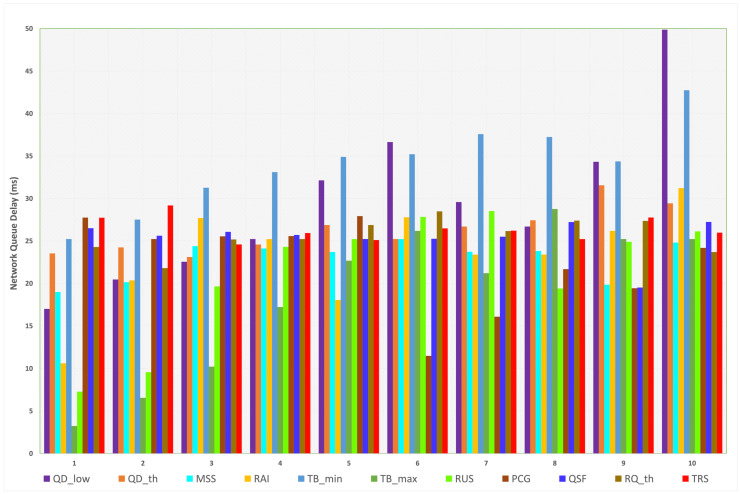
Measured network queue delay for each parameter (L4S/ECN disabled).

Without using L4S/ECN, the network queue delay is depicted in Figure 5. If L4S/ECN is disabled, the default delay value is 25.22 ms. We can note that the overview of the data imposes that increasing the values of the investigated parameters results in higher delay values where the maximum delay value is noticed at the last transition value when $QD_{low}$ equals 1.0. Delay values of $QD_{low}$ and $TB_{min}$ are mostly higher than the rest. The minimum delay value is 3.23 ms, achieved when $TB_{max}$ is at the first transition value (10). The minimum and maximum delays for each parameter are shown in Table 6.

For $QD_{low}$, SCReAM achieved a lower delay when the parameter values were lower than the default value (36.64 ms), except for the last case, in which the delay was at its maximum value (49.87 ms), which indicates that the lowest queue delay target value can be reduced to achieve lower delays. For $QD_{th}$, higher delay values are noticed when the parameter value increases beyond the default value and approximately comparable and close delay values when its value is lower than the default value. Thus, setting the threshold lower than the default value is preferred to enhance congestion detection.

For MSS, lower delay values than the default value are observed in all transitions. The lowest delay values are noticed when MSS is set to 900, 1000, and 1350 bytes. Hence, the Maximum Segment Size can be set at any value within the specific margins to achieve lower delays. Setting RAI higher than the default value results in a higher delay due to extending the process of adjusting the rate over longer periods, while reducing the value achieves an improved delay. Although reducing this value by 75% results in the lowest delay, it causes the algorithm to adjust the rate rapidly, which affects other network metrics. Setting the minimum target bitrate ($TB_{min}$) at 1 Mbps produces the lowest delay because increasing the parameter value above the default value creates additional load on the algorithm so that it does not drop lower than the specified target bitrate. Likewise, when $TB_{max}$ is set at higher values, a higher target bitrate is expected from the algorithm; thus, the network delay grows proportionally.

Controlling the ramp-up speed of the target bitrate is specified by RUS. Increasing or decreasing this parameter does not imply that network performance is enhanced because a high ramp-up speed value can be set, which keeps the network in congestion mode. Reducing the value of this parameter results in lower delays; however, it negatively affects other network performance metrics (such as network throughput, congestion window, and bytes in flight). Thus, certain parameter values can be picked to derive the appropriate network performance. For example, a parameter value of 16 Mbps seems to achieve the most convenient performance by considering all network metrics. Specifying the PCG value allows for composing a suitable pre-congestion guard. Mainly, higher parameter values than the default value result in lower delays. However, it might reduce the accuracy of predicting congestion since the traffic can reach a peak in a short period.

In most transition values of QSF when experimenting with the queue size factor, the delay seems primarily stable, and the difference is very low. Only the parameter value of 0.4 made a noticeable reduction in the delay (22.6%), which also affects network throughput (as will be presented later). Increasing the RTP queue delay threshold value ($RQ_{th}$) carries out higher delays (except the last transition value); lower parameter values reduce the delay because this parameter contributes to the amount of reduction in the target rate. The overall SCReAM performance indicates that the default value and its half (0.05 s) achieve the most appropriate outcome. Finally, TRS is set to reduce the target rate by 5% when the RTP queue delay value exceeds the specified threshold. Although lowering the target rate by 20% reduces the delay slightly, it is unsuitable for losing 20% of the target rate. Thus, using values of 0.95 and higher is only proper.

**Figure 6:**
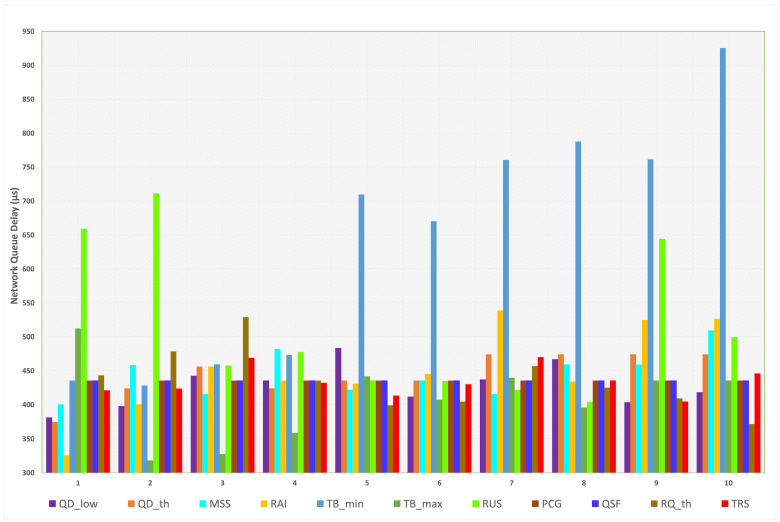
Measured network queue delay for each parameter (L4S/ECN enabled).

Correspondingly with the first case, as shown in Figure 6, when L4S/ECN is enabled, it can be observed that the overall delay is significantly reduced (from ms to µs). The default delay value for this scenario is 435 µs. Most parameters’ values achieved delays between 400 µs and 500 µs. The minimum and maximum delays are 318 µs and 925 µs, respectively. The minimum delay is achieved when the value of $TB_{max}$ is 15 Mbps, which indicates that lowering $TB_{max}$ results in an enhanced network delay. However, it affects network throughput, as will be presented later. The maximum delay is produced when the minimum target bitrate is increased to 10 Mbps, which indicates an increased load in the algorithm due to overhead from increasing the minimum target bitrate. The minimum and maximum delays for each parameter are presented in Table 7.

For $QD_{low}$, when the low queue delay target value is set between 0.05 and 0.15 s, the network delay is close to the default delay; otherwise, the delay is lower. However, reducing the value below 0.05 s can impose a negative performance on other metrics. When the transition values of $QD_{th}$ cross beyond the default value, the delay is constant at 474 µs. The delay is reduced significantly when the first transition value is applied. The effect of MSS indicates that the delay increases when MSS is set beyond 1250 bytes. The lowest delay is noticed when MSS equals 900 bytes.

For RAI, the delay is reduced when the interval is below 15 s. Transition values beyond the default value indicate higher delays. Increasing the value of $TB_{min}$ and $TB_{max}$ beyond the default value will force the algorithm to keep the minimum and maximum target bitrate above the specified value, which increases network delay. For RUS, decreasing or highly increasing the value leads to more congestion as the ramp-up process is slower; hence, the delay increases. When the ramp-up speed is slightly increased (10–16 Mbps), the network delay is reduced to 400 µs.

It is shown that PCG and QSF did not impact SCReAM’s performance, as all transition values resulted in 435 µs (the default value). Thus, increasing or decreasing the congestion guard and the queue size factor will not affect the network delay when L4S/ECN is activated. Setting the RTP queue delay threshold ($RQ_{th}$) higher than the default value will result in a lower delay while reducing it below the default value increases the delay. For TRS, all scale values increase the delay except when 2.5% is used, which reduces the delay to 404 µs.

#### 4.1.2. sRTT

**Figure 7:**
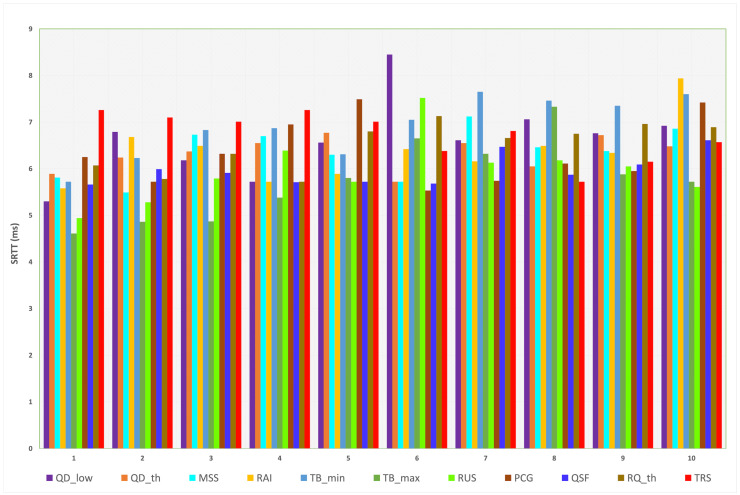
Measured sRTT for each parameter (L4S/ECN disabled).

The effect of each parameter’s transition values on sRTT without using L4S/ECN is illustrated in Figure 7. The default, minimum, and maximum sRTT values are 5.72, 4.61, and 8.45 ms, respectively. The minimum delay is caused by $TB_{max}$, while the maximum is caused by $QD_{low}$. Other parameters’ delays are presented in Table 8. Thus, lowering the margin of the queue delay target and increasing the maximum target bitrate are the most influential parameters on sRTT, negatively and positively. Since sRTT represents the time required for the packet to be transmitted and acknowledged and this metric depends mainly on the physical layer, the data are not expected to change significantly. Thus, we can observe from Figure 7 that most sRTT values fall between 5 and 7 ms.

Although many parameters achieved a lower sRTT than the default value, for all parameters except RUS, the default value seems to gain a more suitable performance. sRTT is reduced only when the $QD_{low}$ value is equal to or lower than 10% of the default value. When MSS is only reduced at 1000 bytes, sRTT is reduced by 4%, as compared to the default value. For RAI, selecting the minimum value (5 ms) of the rate adjust interval can decrease the sRTT to 5.58 ms. Although setting the maximum target bitrate ($TB_{max}$) at the default value achieves a lower sRTT, it limits the algorithm from reaching higher throughput. Thus, 100 Mbps is more efficient. Increasing the ramp-up speed (RUS) to higher than the default value decreases the sRTT; however, it can achieve better performance.

**Figure 8:**
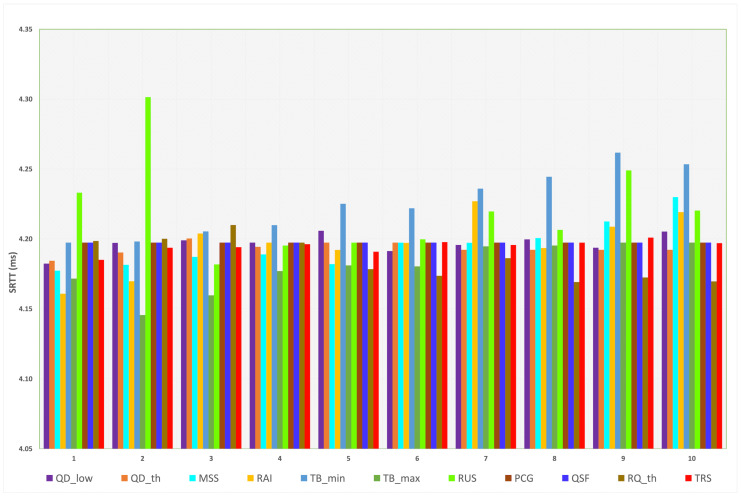
Measured sRTT for each parameter (L4S/ECN enabled).

When L4S/ECN is enabled, the measured sRTT for all parameters is illustrated in Figure 8. The minimum and maximum sRTT for each parameter are depicted in Table 9. When the default value is applied, the sRTT equals 4.197 ms, while the minimum and maximum sRTT are 4.146 ms and 4.3 ms, respectively, produced by different target bitrate transition values. It is shown that when L4S/ECN is enabled, sRTT varies slightly compared to other metrics. Thus, the effect of each parameter when applying several transition values does not impose a high impact on sRTT.

We noticed that specifying threshold values ($QD_{th}$) higher than the default value indicates that the sRTT is reduced to 4.192 ms. A lower MSS than the default value decreases the sRTT. The rate adjustment interval (RAI) can be specified between 5 and 30 ms (based on the target application) to achieve a lower sRTT than the default value. Although increasing the minimum and maximum target bitrates ($TB_{min}$ and $TB_{max}$) indicates a higher sRTT, the maximum target bitrate is preferred to be set at higher values to allow the algorithm to perform at full capacity. Corresponding with the previous metric, PCG and QSF did not impact SCReAM’s performance as all transition values resulted in 4.197 ms (the default value). Higher thresholds of the RTP queue delay ($RQ_{th}$) and lower scales of the target (TRS) decrease the sRTT.

#### 4.1.3. Throughput

**Figure 9:**
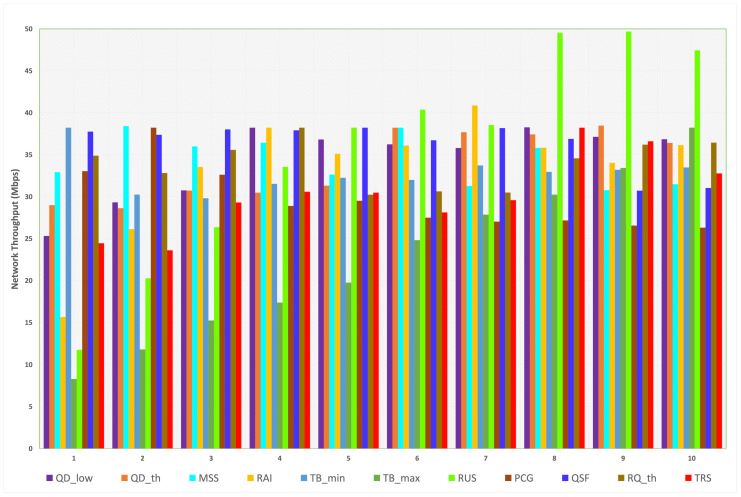
Measured network throughput for each parameter (L4S/ECN disabled).

In this scenario, we will discuss the outcome of each parameter’s transition values on the network throughput when L4S/ECN is disabled. Based on Figure 9, we realize that the obtained throughput values are mostly distributed between 25 and 40 Mbps. The default value for the throughput is 38.23 Mbps, while the minimum and maximum values are 8.29 Mbps and 49.69 Mbps, respectively. We can observe that the maximum throughput is found when we tuned RUS, while the minimum throughput is realized by $TB_{max}$. Thus, when the maximum target bitrate is set to 100 Mbps and the ramp-up speed to 18 Mbps, SCReAM performs at full network capacity. Furthermore, since we are testing network throughput at this stage, the most effective parameter is $TB_{max}$ because it controls how much capacity the algorithm can utilize. The overall minimum and maximum throughput values for each parameter are illustrated in Table 10.

The maximum throughput resulting from the default values of $TB_{min}$, PCG, QSF, $RQ_{th}$, and TRS is due to setting the default maximum target bitrate at 100 Mbps. For the other parameters, increasing the low target queue delay ($QD_{low}$) beyond 50 ms demonstrates slight changes in throughput (35.8–38.27 Mbps). For $QD_{th}$, the queue delay trend threshold can be set at values equal to or higher than 200 ms; lower values result in decreased throughput. Reducing MSS to 1000 bytes produces higher throughput while increasing the adjusted rate interval (RAI) to 35 ms results in a throughput of 40.88 Mbps. $TB_{max}$ caused a stable increase in the throughput when we increased the transition values. At the same time, the minimum throughput achieved (8.29 Mbps) over all of the parameters was found when $TB_{max}$ is 10% of its default value (10 Mbps). RUS has a maximum positive impact over all other parameters since the highest throughput (49.69 Mbps) is reached when RUS is increasing 60% over the default value. For PCG, the maximum value of the throughput (38.23 Mbps) was found at the default value (0.1). After the default value, the throughput gradually decreases until it reaches the minimum value (25.72 Mbps) at the highest transition value of the PCG.

**Figure 10:**
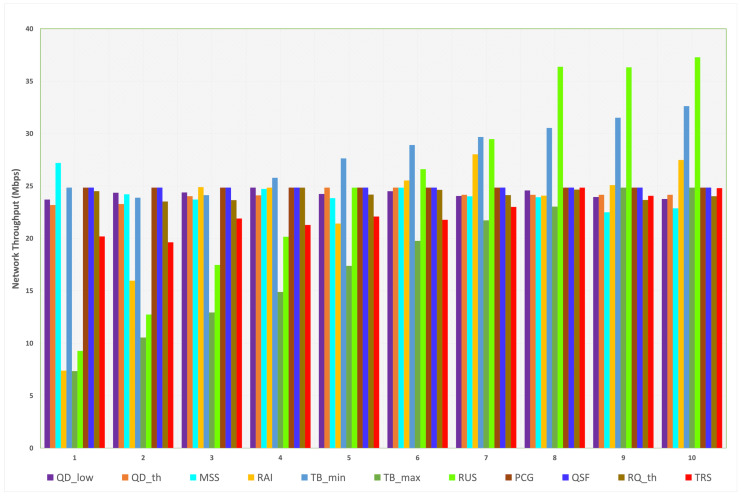
Measured network throughput for each parameter (L4S/ECN enabled).

When L4S/ECN is enabled, the measured throughputs for all parameters are illustrated in Figure 10. The minimum and maximum throughputs for each parameter are depicted in Table 11. The default throughput is 24.84 Mbps (35% less than the L4S/ECN-disabled scenario), while the minimum is 7.35 Mbps and the maximum is 37.29 Mbps. In Table 11, we notice that the minimum throughput value was found due to the impact of $TB_{max}$, and the maximum throughput value was found due to the effect of RUS.

$QD_{low}$, $QD_{th}$, and $RQ_{th}$ had negligible effects on SCReAM’s throughput in the range 23.18–24.84 Mbps while keeping the maximum throughput at the default throughput value (24.84 Mbps). Notice that the $RQ_{th}$ behavior is unstable since the throughput increases and decreases each time the transition value increases. For MSS, the maximum throughput (27.20 Mbps) is at the first transition value (900 bytes), decreasing with the other transition values.

For RAI, we can observe a considerable increase in the throughput since it starts from (7.39 Mbps) at the beginning of the transition values and then doubles at the second value. After that, it continues increasing until the throughput reaches 400% of the first value to reach 27.49 Mbps at the last transition value. For the $TB_{min}$, $TB_{max}$, RUS, and TRS, the throughput increases almost smoothly when we increase the transition values. With a retrieval note, the minimum and maximum throughput values were found at $TB_{max}$ and RUS, respectively. Finally, PCG and QSF did not show any impact on the throughput for all transition values.

### 4.2. Part 2

**Figure 11:**
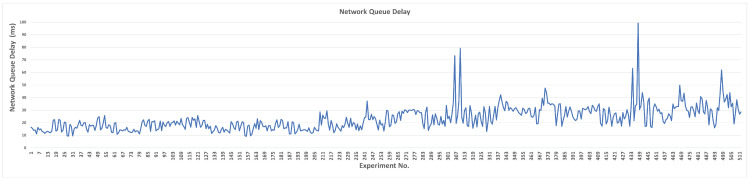
Measured network queue delay for a mixed parameter tuning scenario (L4S/ECN disabled).

**Figure 12:**
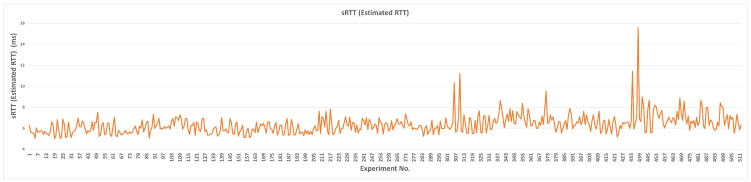
Measured sRTT delay for a mixed parameter tuning scenario (L4S/ECN disabled).

**Figure 13:**
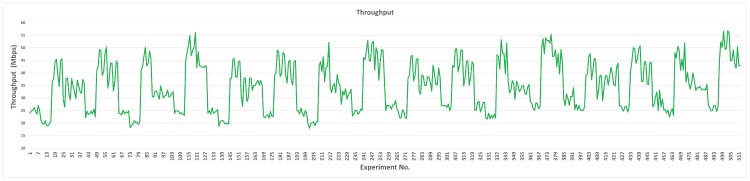
Measured network throughput for a mixed parameter tuning scenario (L4S/ECN disabled).

In the mixed scenario (hybrid experiment phase), we aim to achieve the optimum performance of SCReAM using the previously selected pair values. The minimum network queue delay, sRTT, and maximum throughput are investigated altogether. We will discuss each case (L4S/ECN-disabled and -enabled) separately. When the L4S/ECN is disabled, the network queue delay, sRTT, and throughput are depicted in Figure 11, Figure 12, and Figure 13, respectively.

The following remarks are realized for the network queue delay and sRTT: 329 of 512 experiments achieved lower delays than the original design of SCReAM (25.22 ms). In six experiments, we could even reach delay values below 10 ms. Lower delays are noticed in the first half $QD_{low}$ = 0.025) as compared to the second half ($QD_{low}$ = 0.075). Both threshold ($QD_{th}$) values achieved the lowest delay in our experiments; the difference between both values is 1.4%. Spikes are noticed at the lower values of MSS and PCG and high values of RAI and RUS.

Delays higher than 60 ms are observed in experiments 306, 310, 434, 438, and 498 due to a change in the QSF and TRS values. For the pair values of $RQ_{th}$, the average delay of the first value is higher. Spikes in delay occurred in both values of QSF; higher spikes are noticed at 0.25. Although the higher value of TRS results in high delays in some cases, it can achieve very low delays when certain combinations of parameter sets are used. Overall, higher delays are noticed when PCG = 0.1, QSF = 0.25, and $RQ_{th}$ = 0.05.

The average network queue delay for the total experiments in this scenario is 23.14 ms, indicating that the median is still lower than the original delay. The minimum network queue delay is 9.24 ms, and the parameters’ values are illustrated in Table 12. The sRTT correlates similarly to the network queue delay, except it is between 5.01 and 15.56 ms. Similar to the network queue delay, the highest sRTT values are noticed in the same experiments. However, the lowest sRTT is realized when PCG is lower and TRS is higher. The average sRTT in all experiments is 6.36 ms, and the minimum sRTT is 5.01 ms; the parameters’ values are illustrated in Table 12.

On the other hand, network throughput is described as follows: We noticed a consistent behavior occurring every certain number of experiments where network throughput drops significantly. For example, in experiment 41, network throughput dropped by 39%, caused by updating the values of PCG, QSF, $RQ_{th}$, and TRS. The lowest network throughput is 17.93 Mbps caused due to the following set: $QD_{low}$ = 0.025, RAI = 25, RUS = 8, PCG = 0.5, QSF = 0.1, $RQ_{th}$ = 0.05, and TRS = 1.

Higher throughput is generated when MSS, RAI, and RUS are increased. The most influential parameter on network throughput is PCG; it is noticed that the throughput drops significantly when PCG is equal to 0.5. The average network throughput is 34.58 Mbps, while the maximum network throughput is 56.82 Mbps; the parameters’ values are illustrated in Table 12. Our experiments managed to increase network throughput by 48.6% as compared to the maximum throughput generated by the original design.

To obtain a better understanding of SCReAM’s performance limitations, we conducted additional experiments to demonstrate the typical worst-case scenarios in both modes, shown in Table 13 and Table 14, respectively. In the case where L4S/ECN is disabled, we can notice that the maximum average network queue delay was 99.30 ms, which is increased by 306% compared with the original design’s default value (24.45 ms). In contrast, the maximum average sRTT was 15.56 ms for the same parameters’ setting, which increased by 142% compared with the original design’s default value (6.42 ms). For the average throughput, the minimum recorder value was 17.93 Mbps, which decreased by 51.5% compared with the original design’s default value (37.05 Mbps). When the L4S/ECN is enabled, we can observe that the maximum average network queue delay was 1.856 ms, which is increased by 326% compared with the default value (435.57 µs) of the original design. In comparison, the maximum average sRTT was 4.375 ms for the same parameters’ setting, which increased by 4.16% compared with the default value (4.197 ms) of the original design. For the average throughput, the minimum achieved value was 17.63 Mbps, which decreased by 29% compared with the default value (24.84 Mbps) of the original design.

**Figure 14:**
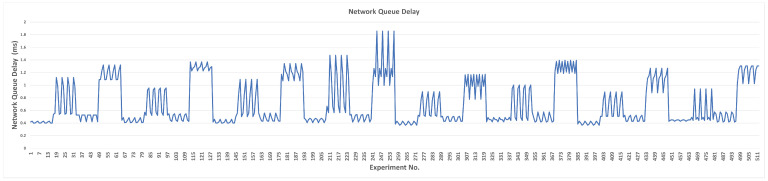
Measured network queue delay for a mixed parameter tuning scenario (L4S/ECN enabled).

**Figure 15:**
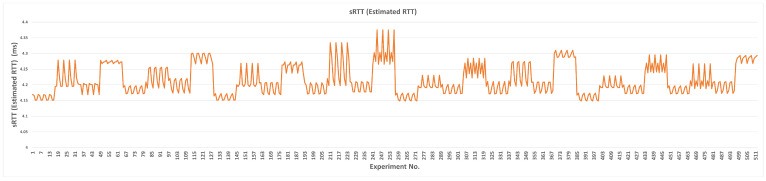
Measured sRTT delay for a mixed parameter tuning scenario (L4S/ECN enabled).

**Figure 16:**
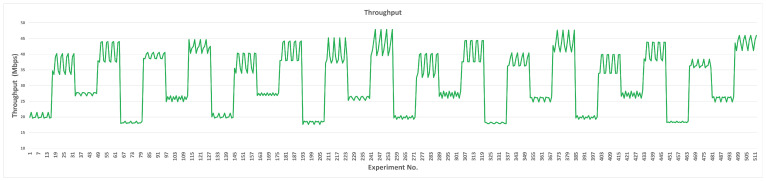
Measured network throughput for a mixed parameter tuning scenario (L4S/ECN enabled).

In the case of the L4S/ECN-enabled, the network queue delay, sRTT, and throughput are depicted in Figure 14, Figure 15, and Figure 16, respectively. The metrics’ values that achieve the optimum performance are illustrated in Table 15. For the network queue delay and sRTT, we can notice that 132 experiments achieved lower network queue delays than the original design of SCReAM (0.435 ms). In comparison, 224 experiments achieved lower sRTT delays than the original value (4.197 ms). In addition, the delays of SCReAM are similar in every four consecutive experiments (the repetitive behavior is shown in Figure 14 and Figure 15) because the PCG and QSF are changing every four experiments. Still, they do not impact SCReAM performance when the L4S/ECN is used. Moreover, lower delays are noticed in the first half (RUS = 8) compared to the second half (RUS = 20). In the same way, network queue and sRTT are lower when RAI = 25 compared to RAI = 35.

The network queue delay is four times higher than the default values (1.856 ms) in experiments 244, 248, 252, and 256 because the $QD_{low}$ has been updated. In the same experiments, the maximum sRTT delay was recorded (4.375 ms), which is 4% more than the original delay. The average network queue delay for the experiments is 0.71 ms, while the sRTT is 4.22 ms. The network queue delay in the first 65 experiments is lower when $RQ_{th}$ is set to 0.05. Notice that both of the parameters $TB_{min}$ = 2 Mbps and $TB_{max}$ = 100 Mbps are fixed in all experiments since they are related, as previously mentioned. Finally, higher network queue and sRTT delays are discovered when $QD_{low}$ = 0.025, $QD_{th}$ = 0.3, MSS = 1200, RAI = 35, RUS = 20, $RQ_{th}$ = 0.3, and TRS = 1.

Experiments 194, 198, 202, and 206 showed minimum throughput (17.6347 Mbps), which is 30% lower than the original throughput (24.84 Mbps). This reduction mainly happened because RUS was reduced from 20 to 8 Mbps. Increasing RAI also impacts the throughput positively. The average throughput for all 512 experiments in this scenario is 31.41 Mbps, which is 27% greater than the original value. On the other hand, the maximum throughput (47.90 Mbps) achieved in our experiments is 93% higher than the original (24.84 Mbps).

## 5. Conclusions

SCReAM is one of the common algorithms to tackle network congestion in a hybrid aspect by combining delay and loss-based schemes. The SCReAM algorithm consists of several parameters that can be investigated to determine their effects on the algorithm and improve its performance. To the best of our knowledge, this is the first study that focuses on exploring SCReAM deeply. In this paper, we propose a guided parameters’ tuning approach to achieve the optimum performance of the SCReAM algorithm through an optimal parameter set in an emulated 5G environment in terms of network queue delay, sRTT, and network throughput. Our approach includes three phases: the initialization phase, the standalone experimentation phase, and the hybrid experimentation phase. The first phase comprises initializing the SCReAM environment for our experiments. The second phase of this study is to investigate each parameter separately to provide clear guidelines on the performance of SCReAM by experimenting with various values for the selected parameters. The third phase focuses on realizing the optimal performance by mixing pair values of each parameter. Two algorithms are designed to generate separate and mixed sets for our experimentation phases. Finally, based on the experiments, we achieved the lowest network queue delay, lowest sRTT, and highest throughput.

As a result, it is important to consider the following guidelines when SCReAM is implemented in a specific domain where the impact of certain parameters can be noted:

1. Single modification (L4S/ECN disabled):
  - Network queue delay and sRTT can be reduced by 87.19% and 19.4%, respectively, when $TB_{max}$ is set at a minimum value (10 Mbps); however, this affects network throughput negatively, where it drops by 45.6% compared to the original value used in SCReAM design (15.24 Mbps).
  - When $TB_{max}$ is set at 100 Mbps (the value we set as the default in our experiments), we noticed that network throughput increased by 150%.
  - The same applies for RUS, where it reduced network queue delay and sRTT by 71% and 14%, respectively.
  - Setting RUS at a high value forces the algorithm to increase the throughput quickly. For example, at 18 Mbps, network throughput increased by 226% compared to the original values in the SCReAM design.
2. Single modification (L4S/ECN enabled):
  - RAI and $TB_{max}$ achieved the lowest network queue delay and sRTT.
  - $TB_{min}$ and RUS resulted in the highest network throughput.
  - PCG and QSF did not impose any effect on all metrics.
  - RAI and $TB_{max}$ managed to reduce network queue delay by 25.23% and 26.94%, respectively.
  - Although RAI and $TB_{max}$ reduced the sRTT slightly, this indicates that when L4S/ECN is used, the impact of any parameter is <2%.
  - $TB_{min}$ and RUS increased the throughput by 31.3% and 50.1%, respectively.
3. Multiple modifications (L4S/ECN disabled):
  - Higher network delays are noticed when modifying the values of PCG, QSF, and $RQ_{th}$.
  - The lowest sRTT is realized when PCG is lower and TRS is higher.
  - The most influential parameter on network throughput is PCG; it is noticed that the throughput drops significantly when its value is equal to 0.5.
4. Multiple modifications (L4S/ECN enabled):
  - PCG and QSF do not impact SCReAM’s performance.
  - Network queue delay and sRTT are lower when specifying lower values of RAI.
  - On the other hand, increasing the RAI results in higher throughput values.
  - Reducing the value of RUS can cause a high reduction in throughput. For example, reducing its value to 8 Mbps causes 30% lower throughput than the original SCReAM default throughput (24.84 Mbps).

In order to illustrate the importance of this study, previous studies that consider SCReAM discussed alternative directions, such as focusing on the challenges of implementing L4S in 5G networks where SCReAM is used as a scalable congestion control algorithm in the network since the evaluation is performed for Augmented Reality (AR) video gaming traffic [36]. Another study performed a comparison of three congestion control algorithms under RTP Media Congestion Avoidance Techniques (RMCAT), including SCReAM, Google Congestion Control (GCC), and Network Assisted Dynamic Adaptation (NADA) [42]. SCReAM achieved lower link capacity utilization; however, SCReAM produced the lowest queue occupation. The authors in [43] presented a modification to their previous FSE-NG algorithm [44] by incorporating SCReAM in specific scenarios to reduce end-to-end delays while increasing RTP throughput. Another experiment of SCReAM on remote-operated working machines was conducted in [45] to evaluate this algorithm based on the quality of video feedback to achieve high video throughput and low latency. Our study proposes the initial guidelines based on our guided parameters’ tuning approach to investigate SCReAM itself as it can be employed in different target applications. Furthermore, we investigated the effect of each parameter in isolation based on QoS metrics. Finally, using the proposed hybrid experimentation model, we achieved the optimum performance based on each metric: network queue delay, sRTT, and network throughput.

Since SCReAM is one of the well-known congestion control algorithms nowadays, it is important to understand its complex design and components. This study presents an initial experimental exploration and optimization approach, which can be utilized in different environments and opens the door for other optimization approaches through the constructed dataset. In the future, we plan to optimize SCReAM by investigating different machine-learning approaches. In addition, it is important to explore and analyze the effect of our experiments on other metrics, such as congestion window and bytes in flight. Furthermore, an efficient video encoding mechanism is required to enhance SCReAM’s performance.

## Figures and Tables

**Table 1:** ECN/L4S bits meaning and codepoints.

Binary Codepoint	Codepoint Name	Meaning
00	Not-ECT	Not ECN-capable transport
01	ECT(1)	ECN-capable transport/ L4S-capable transport
10	ECT(0)	ECN-capable transport/ Not L4S-capable transport
11	CE	Congestion Experienced

**Table 2:** Investigated parameters, definition, default value, and specified margins [10,11].

Abbreviation	Parameter Name	Meaning	Default Value	Margins
$QD_{low}$	QDELAY_TARGET_LO	Target value for the minimum queue delay	0.1 s	0.01–0.2
$QD_{th}$	QDELAY_TREND_TH QDELAY_TREND_LO	Threshold for the detection of incipient congestion	0.2	0.01–0.5
MSS	Maximum Segment Size	Maximum Segment Size (RTP packet size)	1200 byte	900–1400
RAI	RATE_ADJUST_INTERVAL	Interval between media bitrate adjustments	20 ms	5–50
$TB_{min}$	TARGET_BITRATE_MIN	Minimum target bitrate in bps (bits per second)	1 Mbps	1–10
$TB_{max}$	TARGET_BITRATE_MAX	Maximum target bitrate in bps (bits per second)	20 Mbps	10–100
RUS	RAMP_UP_SPEED	Maximum allowed rate increase speed	10 Mbps	2–20
PCG	PRE_CONGESTION_GUARD	Guard factor against early congestion onset	0.1	0–0.9
QSF	TX_QUEUE_SIZE_FACTOR	Guard factor against RTP queue buildup	0.2	0.025–0.5
$RQ_{th}$	RTP_QDELAY_TH	RTP queue delay threshold for a target rate reduction	0.1 s	0.025–0.3
TRS	TARGET_RATE_SCALE_RTP_QDELAY	Scale factor for target rate when RTP queue delay threshold exceeds RTP_QDELAY_TH	0.95	0.7–1.0

**Table 3:** Selection of parameters’ pair values.

Parameter	Pair Values
$QD_{low}$	[0.025, 0.075]
$QD_{th}$	[0.2, 0.3]
MSS	[1000, 1200]
RAI	[25, 35]
RUS	[8, 20]
PCG	[0.1, 0.5]
QSF	[0.1, 0.25]
$RQ_{th}$	[0.05, 0.3]
TRS	[0.95, 1.0]

**Table 4:** Iterations of SCReAM parameters.

Parameter	Iteration/No. of Experiments
$QD_{low}$	1/256
$QD_{th}$	1/128
MSS	1/64
RAI	1/32
$TB_{min}$	Fixed
$TB_{max}$	Fixed
RUS	1/16
PCG	1/8
QSF	1/4
$RQ_{th}$	1/2
TRS	1/1

**Table 5:** Transition values for each parameter [10,11].

Parameter	Transition Value									
	**1**	**2**	**3**	**4**	**5**	**6**	**7**	**8**	**9**	**10**
$QD_{low}$	0.01	0.025	0.05	0.06	0.075	**0.1**	0.125	0.15	0.175	0.2
$QD_{th}$	0.01	0.025	0.05	0.1	0.15	**0.2**	0.25	0.3	0.4	0.5
MSS	900	1000	1050	1100	1150	**1200**	1250	1300	1350	1400
RAI	5	10	15	**20**	25	30	35	40	45	50
$TB_{min}$	**1**	2	3	4	5	6	7	8	9	10
$TB_{max}$	10	15	20	25	30	40	50	60	80	**100**
RUS	2	4	6	8	**10**	12	14	16	18	20
PCG	≈0	**0.1**	0.2	0.3	0.4	0.5	0.6	0.7	0.8	0.9
QSF	0.025	0.05	0.1	0.15	**0.2**	0.25	0.3	0.35	0.4	0.5
$RQ_{th}$	0.025	0.05	0.075	**0.1**	0.125	0.15	0.175	0.2	0.25	0.3
TRS	0.7	0.75	0.8	0.825	0.85	0.875	0.9	**0.95**	0.975	1.0

**Table 6:** Min and max network queue delay (ms) (L4S/ECN cisabled).

Parameter	Min		Max	
	**Delay**	**Value**	**Delay**	**Value**
$QD_{low}$	17	0.01	49.87	0.2
$QD_{th}$	23.11	0.05	31.55	0.4
MSS	18.99	900	(25.22)	Default
RAI	10.62	5	31.22	50
$TB_{min}$	(25.22)	Default	42.74	10
$TB_{max}$	3.23	10	28.76	60
RUS	7.28	2	28.52	14
PCG	11.45	0.5	27.92	0.4
QSF	19.51	0.4	27.24	0.5
$RQ_{th}$	21.8	0.05	28.48	0.15
TRS	24.59	0.8	29.18	0.75

**Table 7:** Min and max network queue delay (µs) (L4S/ECN enabled).

Parameter	Min		Max	
	**Delay**	**Value**	**Delay**	**Value**
$QD_{low}$	381.11	0.005	483.37	0.075
$QD_{th}$	374.52	0.01	474.22	≥0.25
MSS	400.33	900	509.21	1400
RAI	325.66	5	538.96	35
$TB_{min}$	428.15	2	925.36	10
$TB_{max}$	318.20	15	512.30	10
RUS	403.99	16	711.31	4
PCG	(435.57)	All	(435.57)	All
QSF	(435.57)	All	(435.57)	All
$RQ_{th}$	371.56	0.3	529.01	0.075
TRS	404.64	0.975	470.02	0.9

**Table 8:** Min and max sRTT delay (ms) (L4S/ECN disabled).

Parameter	Min		Max	
	**Delay**	**Value**	**Delay**	**Value**
$QD_{low}$	5.30	0.01	7.06	0.15
$QD_{th}$	5.89	0.01	6.77	0.15
MSS	5.49	1000	7.12	1250
RAI	5.58	5	7.94	50
$TB_{min}$	(5.72)	Default	7.65	7
$TB_{max}$	4.61	10	7.33	60
RUS	4.94	2	7.52	12
PCG	5.53	0.5	7.49	0.4
QSF	5.66	0.025	6.61	0.5
$RQ_{th}$	(5.72)	Default	7.13	0.15
TRS	(5.72)	Default	7.26	0.7, 0.825

**Table 9:** Min and max sRTT delay (ms) (L4S/ECN enabled).

Parameter	Min		Max	
	**Delay**	**Value**	**Delay**	**Value**
$QD_{low}$	4.182	0.01	4.206	0.075
$QD_{th}$	4.184	0.01	4.200	0.05
MSS	4.177	900	4.230	1400
RAI	4.161	5	4.227	35
$TB_{min}$	(4.197)	Default	4.262	9
$TB_{max}$	4.146	15	(4.197)	≥80
RUS	4.182	6	4.301	4
PCG	(4.197)	All	(4.197)	All
QSF	(4.197)	All	(4.197)	All
$RQ_{th}$	4.169	0.2	4.210	0.075
TRS	4.185	0.7	4.201	0.975

**Table 10:** Min and max throughput (Mbps) (L4S/ECN disabled).

Parameter	Min		Max	
	**Throughput**	**Value**	**Throughput**	**Value**
$QD_{low}$	15.32	0.01	38.27	0.15
$QD_{th}$	28.63	0.025	38.47	0.05
MSS	30.78	1350	38.42	1000
RAI	15.66	5	40.88	35
$TB_{min}$	29.81	3	(38.23)	Default
$TB_{max}$	8.29	10	(38.23)	100
RUS	11.77	2	49.69	18
PCG	26.31	0.9	(38.23)	Default
QSF	30.72	0.4	(38.23)	Default
$RQ_{th}$	30.24	0.125	(38.23)	Default
TRS	23.61	0.75	(38.23)	Default

**Table 11:** Min and max throughput (Mbps) (L4S/ECN enabled).

Parameter	Min		Max	
	**Throughput**	**Value**	**Throughput**	**Value**
$QD_{low}$	23.70	0.01	(24.84)	0.06
$QD_{th}$	23.18	0.01	(24.84)	0.15 and Default
MSS	22.49	1350	27.20	900
RAI	7.39	5	28.03	35
$TB_{min}$	(24.84)	Default	32.62	10
$TB_{max}$	7.35	10	(24.84)	≥80
RUS	9.28	2	37.29	20
PCG	(24.84)	All	(24.84)	All
QSF	(24.84)	All	(24.84)	All
$RQ_{th}$	23.52	0.05	(24.84)	Default
TRS	19.63	0.75	(24.84)	Default

**Table 12:** Optimum performance parameters’ sets (L4S/ECN disabled).

Metric	Value	SCReAM Parameters’ Values										
		${\mathrm{QD}}_{\mathrm{low}}$	${\mathrm{QD}}_{\mathrm{th}}$	MSS	RAI	${\mathrm{TB}}_{\min}$	${\mathrm{TB}}_{\max}$	RUS	PCG	QSF	${\mathrm{RQ}}_{\mathrm{th}}$	TRS
Min NetQD	9.24 ms	0.025	0.3	1000	25	2	100	20	0.5	0.1	0.3	1
Min sRTT	5.01 ms	0.025	0.2	1000	25	2	100	20	0.1	0.1	0.3	0.95
Max Throughput	56.82 Mbps	0.075	0.3	1200	35	2	100	20	0.1	0.25	0.3	0.95

**Table 13:** Performance parameters’ sets for worst case scenarios (L4S/ECN disabled).

Metric	Value	SCReAM Parameters’ Values										
		${\mathrm{QD}}_{\mathrm{low}}$	${\mathrm{QD}}_{\mathrm{th}}$	MSS	RAI	${\mathrm{TB}}_{\min}$	${\mathrm{TB}}_{\max}$	RUS	PCG	QSF	${\mathrm{RQ}}_{\mathrm{th}}$	TRS
Max NetQD	99.30 ms	0.075	0.3	1000	35	2	100	20	0.1	0.25	0.05	1
Max sRTT	15.56 ms	0.075	0.3	1000	35	2	100	20	0.1	0.25	0.05	1
Min Throughput	17.93 Mbps	0.025	0.3	1200	25	2	100	8	0.5	0.1	0.05	1

**Table 14:** Performance parameters’ sets for worst case scenarios (L4S/ECN enabled).

Metric	Value	SCReAM Parameters’ Values										
		${\mathrm{QD}}_{\mathrm{low}}$	${\mathrm{QD}}_{\mathrm{th}}$	MSS	RAI	${\mathrm{TB}}_{\min}$	${\mathrm{TB}}_{\max}$	RUS	PCG	QSF	${\mathrm{RQ}}_{\mathrm{th}}$	TRS
Max NetQD	1.856 ms	0.025	0.3	1200	35	2	100	20	0.1 and 0.5	0.1 and 0.25	0.3	1
Max sRTT	4.375 ms	0.025	0.3	1200	35	2	100	20	0.1 and 0.5	0.1 and 0.25	0.3	1
Min Throughput	17.63 Mbps	0.025	0.3	1200	25	2	100	8	0.1 and 0.5	0.1 and 0.25	0.05	0.95

**Table 15:** Optimum performance parameters’ sets (L4S/ECN enabled).

Metric	Value	SCReAM Parameters’ Values										
		${\mathrm{QD}}_{\mathrm{low}}$	${\mathrm{QD}}_{\mathrm{th}}$	MSS	RAI	${\mathrm{TB}}_{\min}$	${\mathrm{TB}}_{\max}$	RUS	PCG	QSF	${\mathrm{RQ}}_{\mathrm{th}}$	TRS
Min NetQD	365.12 µs	0.075	0.2	1000	25	2	100	8	0.1 and 0.5	0.1 and 0.25	0.3	1
Min sRTT	4.15 ms	0.075	0.2	1000	25	2	100	8	0.1 and 0.5	0.1 and 0.25	0.3	1
Max Throughput	47.90 Mbps	0.025	0.3	1200	35	2	100	20	0.1 and 0.5	0.1 and 0.25	0.3	1

## Data Availability

Data are contained within the article.

## Abbreviations

The following abbreviations are used in this manuscript:

SCReAM	Self-Clocked Rate Adaptation for Multimedia
RTT	Round Trip Time
sRTT	smoothed Round Trip Time
HTTP	Hypertext Transfer Protocol
QoE	Quality of Experience
IETF	Internet Engineering Task Force
RTP	Real-Time Transport Protocol
QoS	Quality of Service
HSTCP	HighSpeed TCP
AIMD	Additive Increase Multiplicative Decrease
STCP	Scalable TCP
BDP	Bandwidth-Delay product
ECN	Explicit Congestion Notification
XCP	eXplicit Control Protocol
LEDBAT	Low Extra Delay Background Transport
LPCC	Low-Priority Congestion Control
VoIP	Voice over IP
OWD	One-Way Delay
OWDmin	minimum One-Way Delay
DCTCP	Data Center Transmission Control Protocol
L4S	Low-Latency Low-Loss Scalable Throughput
TFRC	TCP-Friendly Rate Control
SACK	Selective Acknowledgement
FEC	Forward Error Correction
ECT	ECN Capable Transport
RTO	Re-transmission Timeout
CE	Congestion Experience
fps	frames per second
PDCP	Packet Data Convergence Protocol
AR	Augmented Reality
RMCAT	RTP Media Congestion Avoidance Techniques
GCC	Google Congestion Control
NADA	Network Assisted Dynamic Adaptation
$QD_{low}$	Queue Delay Target Low
$QD_{th}$	Queue Delay Trend Threshold
MSS	Maximum Segment Size
RAI	Rate Adjust Interval
$TB_{min}$	Minimum Target Bitrate
$TB_{max}$	Maximum Target Bitrate
RUS	Ramp-Up Speed
PCG	Pre-Congestion Guard
QSF	Queue Size Factor
$RQ_{th}$	RTP Queue Delay Threshold
TRS	Target Rate Scale
